# Supplementary material for: Identification of photoperiod-induced specific miRNAs in the adrenal glands of Sunite sheep (Ovis aries)
Source: Front Vet Sci. 2022 Jul 22;9:888207. doi: 10.3389/fvets.2022.888207 (PMC9354845; doi:10.3389/fvets.2022.888207)
Supplement: Supplementary file 1 [file Data_Sheet_1.ZIP › Supplementary file/miRDeep2 file.zip!/miRDeep2 file/Novel_Structure/LP42A1/Novel_501.pdf]

Provisional ID : NC_019467.2_18386
Score total : 0.6
Score for star read(s) : -1.3
Score for read counts : 0
Score for mfe : 0.3
Score for randfold : 1.6
Score for cons. seed :
Total read count : 14
Mature read count : 14
Loop read count : 0
Star read count : 0

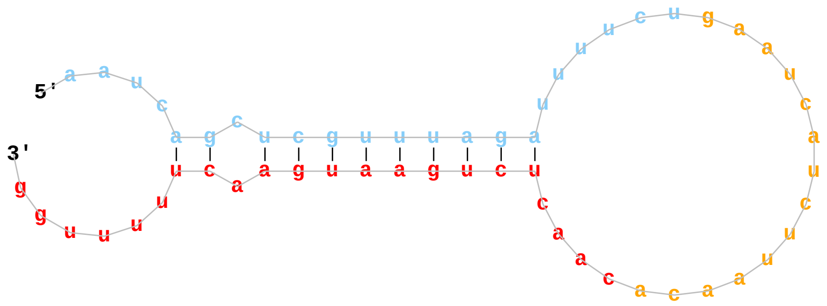

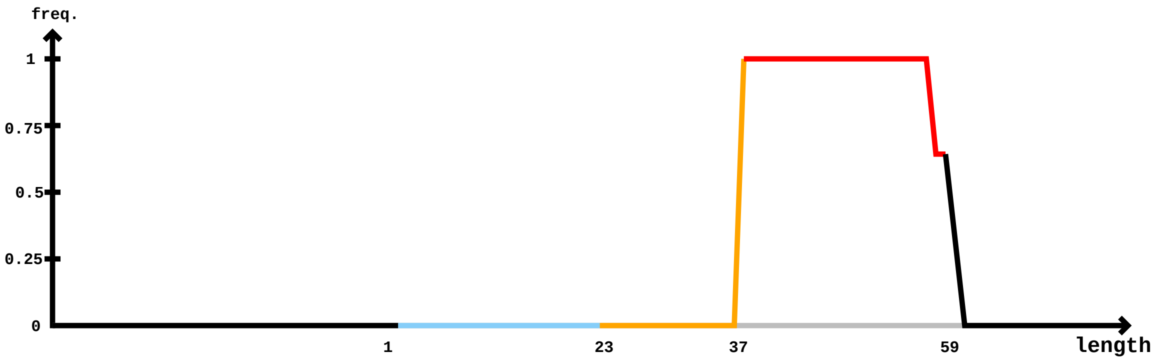

Star Mature

```
5'- uucugagcuugguauuagugauauuggcuuggcuaaucagcucguuuagauuuucugaaucaucuuaacacaacucugaaugaacuuuuuggccagcccaaauacaagcu   -3'    exp
    .....((((((.((((..((.....((((.((((((..((.(((((((((........................))))))))).))..))))))))))))))))))))))    reads    mm    sample
    ......................................................................caacucugaaugaacuuuuu....................    2        0     seq
    ......................................................................Aaacucugaaugaacuuuuu....................    3        1     seq
    ......................................................................Aaacucugaaugaacuuuuugg..................    6        1     seq
    ......................................................................caacucugaaugaacuuuuugg..................    1        0     seq
    ......................................................................Aaacucugaaugaacuuuuuggc.................    2        1     seq
```
